# A Flavone Constituent from *Myoporum bontioides* Induces M-Phase Cell Cycle Arrest of MCF-7 Breast Cancer Cells

**DOI:** 10.3390/molecules22030472

**Published:** 2017-03-15

**Authors:** Jing-Ru Weng, Li-Yuan Bai, Wei-Yu Lin, Chang-Fang Chiu, Yu-Chang Chen, Shi-Wei Chao, Chia-Hsien Feng

**Affiliations:** 1 Department of Marine Technology and Resources, National Sun-Yat-sen University, Kaohisung 804, Taiwan; 2 Division of Hematology and Oncology, Department of Internal Medicine, China Medical University Hospital, Taichung 404, Taiwan; lybai6@gmail.com; 3 College of Medicine, China Medical University, Taichung 404, Taiwan; d5686@mail.cmuh.org.tw; 4 Department of Pharmacy, Kinmen Hospital, Kinmen 891, Taiwan; u8557006@yahoo.com.tw; 5 Cancer Center, China Medical University Hospital, Taichung 404, Taiwan; 6 School of Pharmacy, Kaohsiung Medical University, Kaohsiung 807, Taiwan; wind-chen@yahoo.com.tw; 7 School of Pharmacy, Taipei Medical University, Taipei 110, Taiwan; south102411@gmail.com; 8 Department of Fragrance and Cosmetic Science, College of Pharmacy, Kaohsiung Medical University, Kaohsiung 807, Taiwan; chfeng@cc.kmu.edu.tw

**Keywords:** *Myoporum bontioides*, Myoporaceae, flavone, cell cycle arrest, breast cancer

## Abstract

*Myoporum bontioides* is a traditional medicinal plant in Asia with various biological activities, including anti-inflammatory and anti-bacterial characteristics. To identify the bioactive constituents from *M. bontioides*, a newly-identified flavone, 3,4′-dimethoxy-3′,5,7-trihydroxyflavone (compound **1**), along with eight known compounds, were investigated in human MCF-7 breast cancer, SCC4 oral cancer, and THP-1 monocytic leukemia cells. Among these compounds, compound **1** exhibited the strongest antiproliferative activity with half-maximal inhibitory concentration (IC_50_) values ranging from 3.3 μM (MCF-7) to 8.6 μM (SCC4). Flow cytometric analysis indicated that compound **1** induced G2/M cell cycle arrest in MCF-7 cells. Mechanistic evidence suggests that the G2/M arrest could be attributable to compound **1**’s modulatory effects on the phosphorylation and expression of numerous key signaling effectors, including cell division cycle 2 (CDC2), CDC25C, and p53. Notably, compound **1** downregulated the expression of histone deacetylase 2 (HDAC2) and HDAC4, leading to increased histone H3 acetylation and p21 upregulation. Together, these findings suggest the translational potential of compound **1** as a breast cancer treatment.

## 1. Introduction

Phytochemicals, such as dietary phenolic compounds, phenolic acids, flavonoids, carotenes, and organosulfur, are commonly found in fruits, vegetables, and plants, and have been used in chemoprevention, and as anti-inflammatory, antitumor, antibacterial, and antioxidant agents for centuries [1]. Previous studies suggest that the incidence of cancer could be reduced by the appropriate intake of dietary phytochemicals [2]. More importantly, some phytochemicals have been widely used as therapeutic agents against various diseases [3]. For example, taxol, camptothecin, and vinblastine show antitumor activity against breast, lung, bladder, and other cancers [3]. *Myoporum bontioides* (Myoporaceae) is an evergreen shrub distributed throughout Taiwan, South China, and Japan [4]. The moisturizing property of this plant has attracted considerable interest in its development for further application in the cosmetic industry [5]. In China, *M. bontioides* has been used as a folk medicine for pulpitis and sciatica for a long time [6,7,8].

The members of the family Myoporaceae are known for producing sesquiterpenes, volatile oils, and flavonoids, which have activities against insects, bacteria, inflammation, and cancer [9,10,11,12]. To further explore their antitumor activity, the active constituents of the acetone extract of *M. bontioides* leaves were isolated. In this article, we report the isolation and structural elucidation of a newly-identified flavone, 3,4′-dimethoxy-3′,5,7-trihydroxyflavone (**1**), along with eight known constituents, myoporone (**2**), rhamnocitrin (**3**), norartocarpetin (**4**), 5,7,4′-trihydroxyflavone (**5**), tricin (**6**), diosmetin (**7**), 3,3′-dimethoxyquercetin (**8**), and β-sitosterol (**9**). We investigated the antitumor activities of compounds **1** and **3**–**8** against a panel of human cancer cell lines, and the antitumor mechanism of compound **1** against breast cancer cells. 

## 2. Results

### 2.1. Isolation of Compounds ***1**–**9*** from the Acetone Extract of M. bontioides Leaves

**Figure 1:**
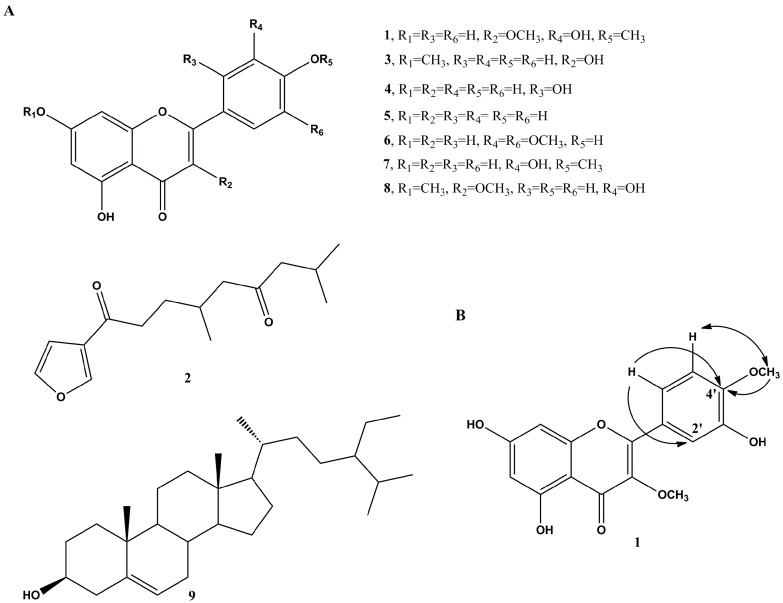
Compounds isolated from *M. bontioides*. (**A**) Chemical structures of compounds **1**–**9**; and (**B**) key HMBCs (H→C) and selected NOESY (H↔H) correlations of compound **1**.

Repeated chromatography of the acetone extract of *M. bontioides* leaves (3.1 kg dry weight) using silica gel yielded compounds **1**–**9** (Figure 1A). High-resolution electron ionization mass spectrometry (HREIMS) data showed a molecular ion peak at *m/z* 330.0743, corresponding to the molecular formula C_17_H_14_O_7_ (calcd., 330.0740). The infrared (IR) spectrum of **1** showed hydroxyl and chelated carbonyl absorption bands at 3372 and 1655 cm^−1^, respectively, while its ultraviolet (UV) spectrum exhibited absorption maxima (209, 255, and 355 nm) consistent with those of a flavone structure [13]. The ^1^H- and ^13^C-NMR spectra (Supplementary Materials) were similar to those of 3,3′-dimethoxyquercetin (**8**) except for C-3′ and C-4′ [14]. The HMBC correlations of OMe-4′/C-4′ and H-6′/C-2′ and C-4′ and the cross-peak of H-5′/OMe-4′ in the NOESY spectrum confirmed that the methoxyl group was linked at C-4′ (Figure 1B). Therefore, compound **1** was characterized as 3,4′-dimethoxy-3′,5,7-trihydroxyflavone.

The chemical investigation also yielded eight known compounds, myoporone (**2**) [15], rhamnocitrin (**3**) [16], norartocarpetin (**4**) [17], 5,7,4′-trihydroxyflavone (**5**) [18], tricin (**6**) [19], diosmetin (**7**) [20], 3,3′-dimethoxyquercetin (**8**) [14], and β-sitosterol (**9**) [21], which were all identified based on the previously published spectroscopic data.

### 2.2. Compound ***1*** Inhibits Growth of MCF-7 Cells

**Figure 2:**
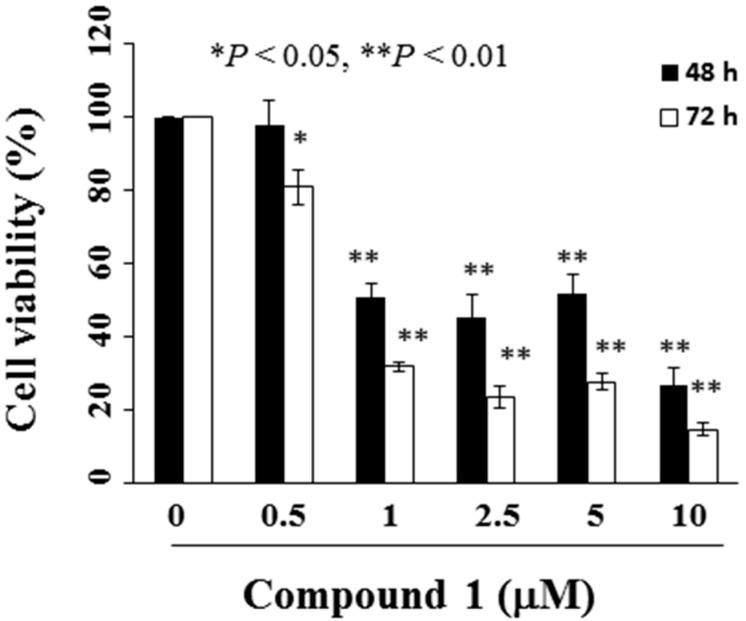
Inhibitory effects of compound **1** on viability of MCF-7 breast cancer cells. Cells were treated with compound **1** at the indicated concentrations for 48 and 72 h, and cell viability was determined by MTT assay. Data are mean ± standard deviation (SD, *n* = 6). * *p* < 0.05 and ** *p* < 0.01 compared to control group.

To assess the potential antitumor activities of these compounds, we examined the antiproliferative effects of compounds **1** and **3**–**8** using the MTT assay in a panel of human cancer cell lines, including MCF-7 breast cancer, SCC4 oral cancer, and THP-1 leukemia cells (Table 1). The antiproliferative effect of compound **2** was not examined because it was unstable in the culture medium. The MTT assay suggests that compound **1** had the strongest antiproliferative activity against all three cancer cell lines among the test compounds. Compound **1** suppressed the viability of MCF-7 breast cancer, SCC4 oral cancer, and THP-1 leukemia cells with 48 h half-maximal inhibitory concentration (IC_50_) values of 3.3 ± 0.6, 8.6 ± 2.7, and 8.5 ± 0.6 µM, respectively. We subsequently focused on characterizing compound **1** because it had the strongest antiproliferative activity among the isolated compounds against MCF-7 cells. The IC_50_ of compound **1** against MCF-7 cell growth was 1.6 µM at 72 h in the MTT assay (Figure 2).

### 2.3. Compound ***1*** Induces G2/M Arrest and Apoptosis in MCF-7 Cells

**Figure 3:**
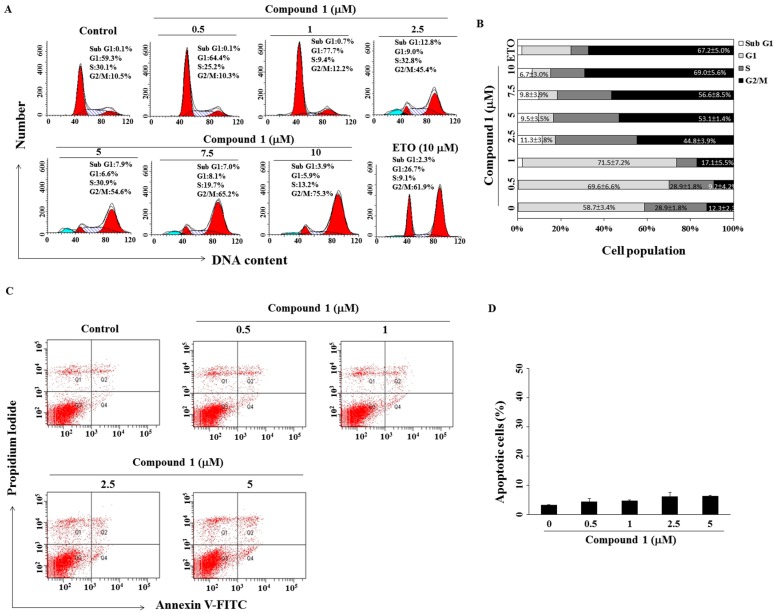
Compound **1** induced G2/M arrest and apoptosis in MCF-7 breast cancer cells. (**A**) Effect of compound **1** on cell cycle distribution. MCF-7 cells were treated with compound **1** at the indicated concentrations for 48 h, followed by propidium iodide (PI) staining and flow cytometric analysis. Treatment with etoposide (ETO) at 10 µM was used as a positive control. Three independent experiments were performed; and data are presented in (**B**) as mean ± standard deviation (SD, *n* = 3); (**C**) the effect of compound **1** on annexin V/PI staining of MCF-7 cells for 48 h; and (**D**) the percentages in the graphs represent the percent of cells in the respective quadrants. Columns, mean; bars, SD (*n* = 3).

To determine whether compound **1** inhibited cell growth by modulating the cell cycle, MCF-7 cells were treated for 48 h and stained with propidium iodide (PI). Flow cytometric analysis of the cell cycle indicated that compound **1** caused G2/M accumulation (Figure 3A,B, etoposide was a positive control). For MCF-7 cells, the cell population in the G2/M phase increased from 12.3% ± 2.3% in the control group to 69.0% ± 5.6% in 10 μM compound **1** group (*p* < 0.005). Although there were occurrences of apoptosis, the cells undergoing apoptosis accounted less than 10% of cells even at the concentration of 5 μM of compound **1** which suggested that apoptosis might not be the major event. (Figure 3C,D).

### 2.4. Compound ***1*** Modulates Cell Cycle-Related Proteins in MCF-7 Cells

**Figure 4:**
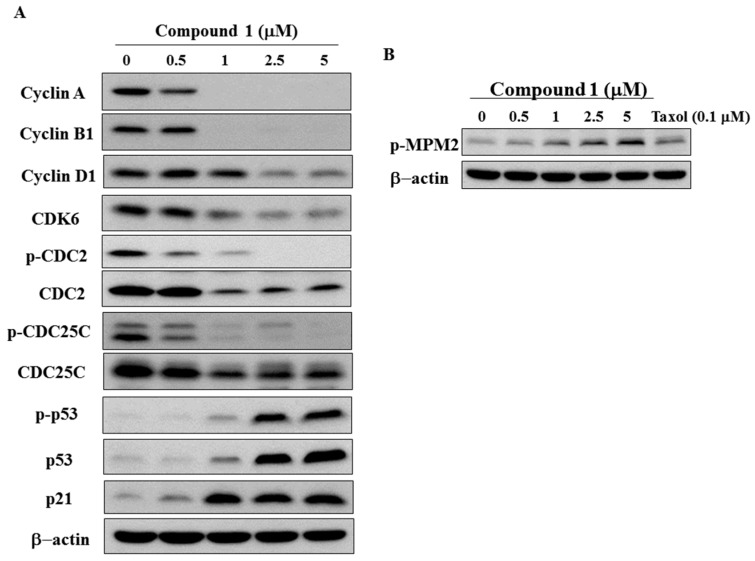
Effects of compound **1** on cell cycle-regulatory proteins. (**A**) Western blotting analysis of the phosphorylation/expression of cell cycle-regulatory proteins, cyclin A, cyclin B1, cyclin D1, CDK6, CDC2, CDC25C, p53, and p21. MCF-7 cells were exposed to compound **1** at the indicated concentrations for 48 h; and (**B**) the effect of compound **1** on the expression of phosphorylated MPM2. Treatment with taxol at 0.1 µM was used as a positive control.

Previous studies showed that dysregulation of cyclins and cyclin-dependent kinases (CDKs) enhances tumor growth [22,23]. To further investigate the anti-tumor mechanism underlying compound **1**-induced G2/M arrest, we evaluated cell cycle-related proteins from the lysates of MCF-7 cells treated with the indicated concentration of compound **1**. Compound **1** downregulated the expression levels of several pivotal cell cycle-regulatory proteins including cyclin A, cyclin B1, cyclin D1, CDK6, total cell division cycle 2 (CDC2), and phosphorylated (p)-CDC2 (Figure 4A). CDC25C, a phosphatase responsible for CDC2 activation, was concurrently downregulated in both the phosphorylated and total form (Figure 4A). A previous report that p53 downregulated transcriptional activity by directly binding to a promoter element of CDC25C [24] prompted us to examine the phosphorylation and expression of p53, as well as its downstream target, p21. Both phosphorylated and total p53 and p21 were upregulated by compound **1** (Figure 4A).

These findings suggest that compound **1** inhibited MCF-7 cell proliferation by modulating cell cycle-related proteins and inducing G2/M arrest. To further determine if the blockade occurred at the G2 or M-step, we examined the expression of p-mitotic protein monoclonal 2 (p-MPM2), which is a mitotic marker [25]. Compound **1** increased p-MPM2 in a dose-dependent manner, suggesting that the M phase was arrested by compound **1** (Figure 4B).

### 2.5. Compound ***1*** Induces HDAC Inhibition in MCF-7 Cells

**Figure 5:**
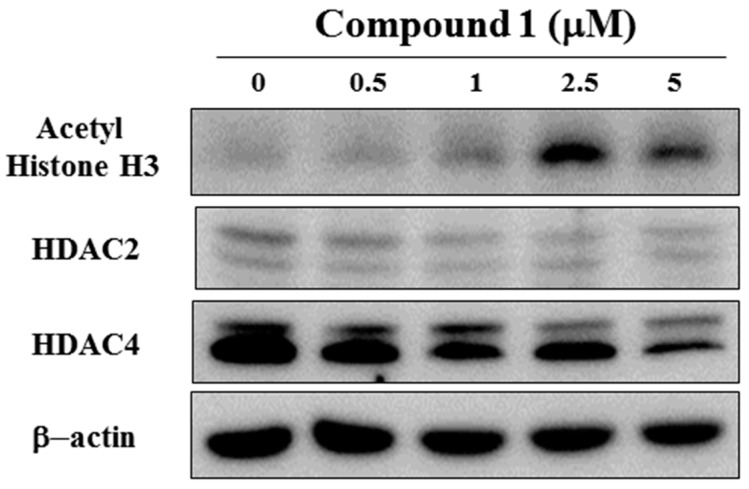
Western blot analysis of the effects of compound **1** on the expression of acetyl histone H3, HDAC2, and HDAC4. MCF-7 cells were exposed to compound **1** at the indicated concentrations for 48 h.

Previous studies showed that flavonoids regulate the activity of histone deacetylases (HDACs), and their inhibition is an epigenetic mechanism for the regulation of the cell cycle and inhibition of cell growth [26,27,28]. To investigate the role of HDACs in compound **1**-induced cell cycle arrest, the protein expression and activity of HDACs were evaluated in MCF-7 cells treated with compound **1** (Figure 5). Treatment with compound **1** decreased the protein expression of HDAC2 and HDAC4, accompanied with an increase in the acetyl form of histone H3 (Figure 5).

### 2.6. Compound ***1*** Increases Reactive Oxygen Species (ROS) Generation in MCF-7 Cells

**Figure 6:**
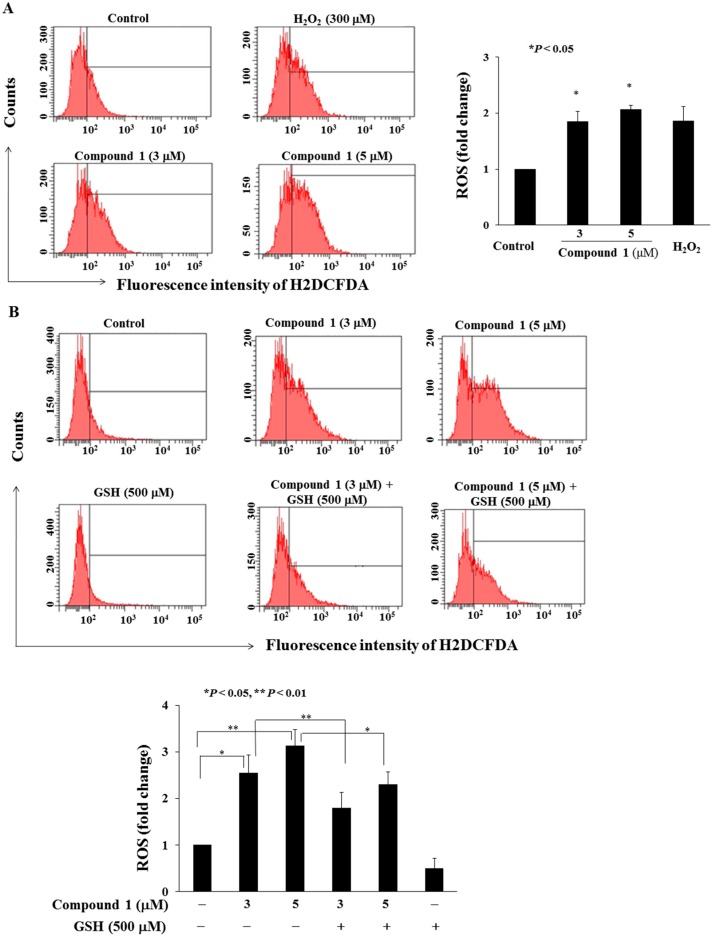
Compound **1** increased reactive oxygen species (ROS) generation. (**A**) Left panel, cells were treated with DMSO or compound **1** at the indicated concentration or 300 μM H_2_O_2_ for 3 h. Right panel, statistical analysis of ROS production in MCF-7 cells. (*n* = 3). * *p* < 0.05 compared to control group; (**B**) Top, pre-treatment with 500 μM glutathione (GSH) reversed compound **1**-induced ROS production; and bottom, statistical analysis of rescued effect of GSH on compound **1**-induced ROS production. Data are mean ± standard deviation (SD, *n* = 3).

Reactive oxygen species (ROS) generation is responsible for the antitumor effect of several phytochemicals, including curcumin, epigallocatechin-3-gallate (EGCG), and resveratrol [29,30,31]. Therefore, we next examined the effect of compound **1** on ROS generation of MCF-7 cells (Figure 6) and found it increased ROS production in a concentration-dependent manner (H_2_O_2_ was the positive control, Figure 6A). Pre-treatment with glutathione (GSH) partially inhibited ROS generation by compound **1** (Figure 6B).

## 3. Discussion

Accumulating evidence indicates that phytochemicals including flavonoids, polysaccharides, saponins, and polyphenolic compounds play important roles in preventing or treating chronic diseases, including cardiovascular diseases, diabetes mellitus, obesity, neurodegenerative diseases, gastrointestinal cancer, and breast cancer [1,32,33,34,35]. Preliminary studies have shown promising results, therefore, numerous polyphenolic compounds and flavonoids have been evaluated for potential antitumor efficacy in ongoing clinical trials [36].

In this study, a new flavone (**1**) and eight known compounds were isolated and identified from *M. bontioides*. Compound **1**, which was characterized for the first time, exhibited a stronger antiproliferative activity against the three human cancer cell lines tested than that of the other eight known constituents. Comparing the IC_50_ values with structures of the individual compounds, we found that replacing a hydrogen at C-3 (i.e., **7**) resulted in a substantial loss of antitumor activity (Table 1). In addition, the compound with a methoxyl group at C-7 (compound **1** vs. compound **8**) showed slightly decreased cytotoxicity against MCF-7 and THP-1 cells. Furthermore, the replacement of a methoxyl group at C-5′ (compound **6** vs. compound **5**) led to specific cytotoxicity against the SCC4 cell line. Moreover, compounds **6** and **7** exhibited lower antitumor activity than compounds **8** and **1** did against MCF-7 cells and, therefore, we speculated that the methoxyl group at C-3 played an integral role in mediating the cytotoxicity. The mechanistic study showed that compound **1** upregulated p53 and p21, downregulated several pivotal cell cycle-regulatory proteins, inhibited HDAC expression, and led to M phase arrest in MCF-7 cells.

G2/M is a cell cycle phase during which the cells prepare, and the chromosome segregates into two daughter cells. The cell cycle propagation is tightly controlled by cell cycle-related proteins including cyclins, CDKs, and CDK kinase inhibitors p21 and p27 [37]. CDC25C, a phosphatase, activates CDC2 by removing both its phosphorylated residues at tyrosine 15 and threonine 14, leading to the onset of mitosis [38,39]. One pivotal key player among the cell cycle-related proteins in the milieu is p53, which has been reported to control the G2/M cell cycle checkpoint under stress signals [40,41,42]. For instance, the upregulation of p53 and p21 causes G2 phase arrest when DNA damage occurs [41,42]. Therefore, it would be reasonable to target p53 as an anticancer strategy. Moreover, several phytochemicals and their derivatives, including indole-3-carbinol, curcumin, and flavonoids have shown inhibition of cancer cell growth through p53 induction [43,44,45].

HDACs, which are epigenetic regulators of histones, participate in signal transduction, apoptosis, cell cycle regulation, and angiogenesis [46]. The relationship between HDACs and cell cycle has been well studied. HDACs deacetylate and regulate the activity of key cell cycle-related proteins, including p53, E2F, and pRb [47]. For example, HDAC2 is recruited to the promoter of p53-dependent target genes as a co-repressor to inhibit their transcription, and HDAC inhibitors can reverse the resistance of antiestrogen therapies in breast cancer [48,49]. Wilson et al. reported that HDAC4 forms a part of the HDAC4-HDAC3-N-CoR/SMRT corepressor complex that represses p21 transcription in maintaining the growth of colon cancer cells [50]. Our study showed that compound **1** modulated cell cycle-regulatory proteins, induced G2/M arrest, and inhibited HDAC expression in MCF-7 cells. We speculate that HDACs are involved in the cell cycle regulation and growth inhibition of MCF-7 cells by compound **1**. Similarly, some phytochemicals, genistein, EGCG, and curcumin showed antiproliferative activities in vitro and in vivo by modulating HDACs [51,52].

In summary, compound **1** modulates HDACs and cell cycle-regulatory proteins, arrests cells in the M phase, increases ROS generation and finally, inhibits MCF-7 cell proliferation.

## 4. Materials and Methods

### 4.1. General

Chromatographic purification and spectroscopic characterization of the test agents were conducted using the following products and instruments. TLC, silica gel 60 F_254_ pre-coated plates (Merck, Darmstadt, Germany); column chromatography (CC), silica gel 60 (70–230 or 230–400 mesh, Merck); UV, Jasco UV-240 spectrophotometer (λ_max_ (log ε) in nm) (Jasco Corporation, Tokyo, Japan); optical rotation, Jasco DIP-370 polarimeter (in chloroform [CHCl_3_]) (Jasco Corporation); Fourier transform IR (FT-IR), Shimadzu-IR Prestige-21 FTIR spectrophotometers (in cm^−1^) (Shimadzu Corporation, Tokyo, Japan); ^1^H-, ^13^C-NMR, and two-dimensional (2D)-NMR Spectra, Agilent Technologies DD2 600 spectrometers (δ in ppm rel. to Me_4_Si as internal standard; *J* in Hz) (Agilent Technologies, Santa Clara, CA, USA), and EIMS and HREIMS, Finnigan Thermo Quest MAT-95XL mass spectrometer [*m/z* (rel. %)] (Thermo Scientific, Waltham, MA, USA).

### 4.2. Plant Material

The leaves of *M. bontioides* (Myoporaceae) were collected and identified by one of the co-authors, Dr. Wei-Yu Lin in Kinmen County, Taiwan in October 2011, and a voucher specimen (2011) has been deposited in the College of Medicine, China Medical University.

### 4.3. Extraction and Isolation

The leaves of *M. bontioides* (3.1 kg) were ground, extracted with acetone at 25 °C, and concentrated under reduced pressure to afford a brown residue (90 g). This residue was fractionated using silica gel column chromatography using *n*-hexane:ethyl acetate (EtOAc), 19:1; *n*-hexane:EtOAc, 9:1; *n*-hexane:EtOAc, 4:1; *n*-hexane:EtOAc, 1:1, and *n*-hexane:EtOAc:methanol (MeOH), 1:1:1, to yield five fractions (A–E). Fractions B and E were further subjected to silica gel column chromatography to obtain compounds **1**–**9** as described below. Fraction E (1.0 g), CH_2_Cl_2_–MeOH (13:1) yielding fractions E_1_–E_6_; fraction E_6_ (238 mg), CH_2_Cl_2_:acetone (4:1) yielding **1** (10 mg) and **2** (5 mg). Fraction E_5_ (172 mg), CHCl_3_:acetone (5:1) yielding **3** (4 mg) and **4** (5 mg). Fraction E_3_ (59 mg), CHCl_3_:MeOH (9:1) yielding **5** (3 mg) and **6** (3 mg). Fraction E_4_ (49 mg), CHCl_3_:EtOAc (9:1) yielding **7** (6 mg). Fraction B (311 mg), *n*-hexane:EtOAc (9:1) providing fractions B_1_–B_6_; fraction B_4_ (105 mg), *n*-hexane–acetone (4:1) yielding **8** (12 mg). Fraction B_3_ (95 mg), *n*-hexane:EtOAc (4:1) yielding **9** (28 mg).

*3,4′-Dimethoxy-3′,5,7-trihydroxyflavone* (**1**): yellow powder; UV (MeOH) λ_max_ (log ε) nm: 209 (4.45), 255 (4.20), 268 (4.20), 295 (3.95), 355 (4.15); (AlCl_3_): 209 (4.47), 268 (4.24), 299 (3.98), 362 (4.11), 401 (4.12); (NaOAc): 219 (4.78), 276 (3.30), 321 (4.00), 374 (4.07); (NaOAc-H_3_BO_3_): 218 (4.77), 256 (4.15), 269 (4.15), 296 (3.90), 358 (4.09); (NaOMe): 212 (4.70), 272 (4.47), 305 (3.98), 389 (4.18); IR (KBr) υ_max_: 3452, 1655 cm^−1^; ^1^H-NMR (CH_3_OH, 600 MHz): δ 3.88 (3H, s, OMe-3), 3.95 (3H, s, OMe-4′), 6.25 (1 H, d, *J* = 2.4 Hz, H-6), 6.51 (1 H, d, *J* = 2.4 Hz, H-8), 7.12 (1 H, d, *J* = 8.4 Hz, H-5′), 7.64 (1 H, d, *J* = 2.0 Hz, H-2′), 7.66 (1H, dd, *J* = 8.4, 2.0 Hz, H-6′); ^13^C-NMR (CH_3_OH, 150 MHz): δ 56.3 (OMe-4′), 60.2 (OMe-3), 94.5 (C-8), 99.4 (C-6), 105.9 (C-10), 112.1 (C-5′), 115.8 (C-2′), 121.8 (C-6′), 124.1 (C-1′), 139.5 (C-3), 147.3 (C-3′), 150.9 (C-4′), 156.5 (C-2), 157.8 (C-9), 163.2 (C-5), 165.0 (C-7), 179.5 (C-4); EIMS (70 eV) *m/z* (rel. int.): 330 [M]^+^ (100), 301 (10), 287 (31); HREIMS *m/z* 330.0743 (calcd. for C_17_H_14_O_7_, 330.0740). The spectra were showed in supplementary materials.

The structures of compounds **2**–**9** were identified using spectroscopic methods and were compared with literature data [14,15,16,17,18,19,20,21].

### 4.4. Reagents

All of the chemicals used were dissolved in dimethyl sulfoxide (DMSO) and were added to the culture medium at indicated concentrations to a final DMSO concentration <0.1%. The following antibodies were used: p-^15^Ser p53, p53, CDK6, CDC25C, p-^216^Ser CDC25C, CDC2, p-^15^Tyr CDC2, cyclin A, cyclin B1, cyclin D1, HDAC2, HDAC4, p21 (Cell Signaling Technologies, Beverly, MA, USA); β-actin (Sigma-Aldrich, St. Louis, MO, USA); p-Ser/Thr-Pro MPM2 (Merck Millipore Corporation, Darmstadt, Germany); and acetyl Histone H3 (Upstate, Temecula, CA, USA). The enhanced chemiluminescence (ECL) system for detection of immunoblotted proteins was from GE Healthcare Bioscience (Piscataway, NJ, USA). All other chemicals and reagents were obtained from Sigma-Aldrich (St. Louis, MO, USA) unless otherwise mentioned.

### 4.5. Cell Culture

MCF-7 breast cancer, SCC4 oral cancer, and THP-1 acute myeloid leukemia cells were purchased from the American Type Culture Collection (ATCC, Manassas, VA, USA). MCF-7 and SCC4 cells were cultured in Dulbecco’s modified Eagle’s medium (DMEM)/Ham’s F-12 medium (Gibco, Grand Island, NY, USA) and THP-1 cells were cultured in Roswell Park Memorial Institute (RPMI)-1640 medium (Invitrogen, Carlsbad, CA, USA). All cells were supplemented with 10% heat-inactivated FBS, 5 mg/mL of penicillin, 10 mg/mL of neomycin, and 5 mg/mL streptomycin at 37 °C in a humidified incubator in an atmosphere of 5% CO_2_.

### 4.6. MTT Assay for Cell Viability

The suppressive effects of the compounds on cell viability were assessed using the MTT assay [53] in six replicates. Cells (5 × 10^3^/200 μL) were seeded in 96-well flat-bottomed plates in 10% FBS-supplemented medium, incubated for 24 h, and then they were exposed to various concentrations of the compounds dissolved in DMSO (final DMSO concentration, 0.1%) in 5% FBS-supplemented medium. Control cells were treated with the DMSO vehicle at a concentration equal to that of the compound-treated cells. Then, the medium was removed, replaced with 200 μL 0.5 mM MTT in 10% FBS-containing DMEM/Ham’s F-12 medium, and the cells were incubated in a 5% CO_2_ incubator at 37 °C for 3 h. After removing the supernatant, the reduced MTT dye was solubilized in DMSO, and the absorbance at 570 nm was determined using a plate reader. The test agent-treated cell viability was expressed as a percentage of the viable control cells. The IC_50_ values of each group were calculated using median-effect analysis and presented as the mean ± standard deviation (SD).

### 4.7. Flow Cytometry Analysis

Cell cycle analysis was performed using flow cytometry [54]. Briefly, MCF-7 breast cancer cells (1 × 10^5^) were plated and treated with compound **1** for 48 h with 5 % FBS-supplemented DMEM/F12. The cells were collected, fixed in 70% cold ethanol for 4 h at 4°C, centrifuged at 1200 rpm for 5 min, and then re-suspended in ice-cold PBS containing 2% FBS. Then, the cells were stained with propidium iodide (PI) and analyzed using flow cytometry and the multicycler (ModFitLT 3.0) software program (Becton Dickinson, Becton, Germany). For apoptosis evaluation, cells were stained with annexin V and PI (1 μg/mL) and determined on a BD FACSAria flow cytometer (Becton Dickinson).

### 4.8. ROS Generation

ROS production was detected using the fluorescence dye 2′,7′-dichlorodihydrofluoresceindiacetate (H2DCFDA, Molecular Probes, Eugene, OR, USA) [53]. The cellular ROS content was detected using flow cytometry according to the manufacturer’s instruction. Briefly, cells (2.5 × 10^5^/mL) were treated with DMSO or compound **1** (0–5 μM) with or without GSH pre-treatment for 15 min for 3 h. Then, the cells were washed twice with PBS and stained with H2DCFDA (5 μM) at 37 °C for 30 min. After washing with PBS, the fluorescence intensity induced by ROS generation was assessed using a flow cytometer (BD FACSCanto II, Becton-Dickinson, Becton, Germany).

### 4.9. Western Blot Analysis

The drug-treated cells were collected, washed with ice-cold PBS, and then resuspended in lysis buffer [53]. Soluble cell lysates were collected after centrifugation at 1500× *g* for 5 min. Equivalent amounts of protein (60–100 μg) from each cell lysate were resolved on 10% SDS-polyacrylamide gels and transferred to nitrocellulose membranes, which were blocked with 5% nonfat milk in PBS containing 0.1% Tween 20 (PBST). Then, the membranes were incubated overnight with the corresponding primary antibodies (1:1000–1:2000) at 4 °C, followed by washing with PBST four times, incubation with the secondary antibody (1:1000) in PBST at room temperature for 1 h, and then they were visualized using ECL.

### 4.10. Statistical Analysis

The data were presented as means ± SD. Statistical analysis was performed using Student's *t-*test for two-group comparisons, and *p-*values < 0.05 were considered statistically significant.

## Figures and Tables

**Table 1:** Antiproliferative activities of compounds **1** and **3–8** against different cancer cell lines.

Compound	IC_50_ (μM) ^a^		
	SCC4 ^b^	MCF-7 ^b^	THP-1 ^b^
**1**	8.6 ± 2.7	3.3 ± 0.6	8.5 ± 0.6
**3**	9.6 ± 2.4	8.9 ± 1.0	> 30
**4**	21.3 ± 4.4	12.7 ± 1.0	18.5 ± 3.7
**5**	>30	10.3 ± 1.0	17.0 ± 1.7
**6**	12.5 ± 1.7	>30	>30
**7**	>30	>30	>30
**8**	9.4 ± 1.0	16.8 ± 3.5	13.5 ± 1.8
**Etoposide *^c^***	2.6 ± 0.4	8.8 ± 1.0	2.4 ± 0.4

^a^ Data are presented as mean ± S.E.M. (*n* = 3–6); ^b^ Key to all cell lines: MCF-7, human breast adenocarcinoma; THP-1, human monocytic leukemia; SCC4, human oral squamous cell carcinoma; ^c^ Etoposide was used as a positive control.

## Supplementary Materials

The ^1^H and ^13^C-NMR, HMQC, HMBC, and NOESY plots of compound **1** are provided in the Supplementary Materials.

## Abbreviations

CDC	cell division cycle
CDK	cyclin-dependent kinase
DMSO	dimethyl sulfoxide
ECL	enhanced chemiluminescence
FBS	fetal bovine serum; HDACs, histone deacetylases
MTT	3-(4,5-dimethylthiazol-2-yl)-2,5-diphenyl-2*H*-tetrazolium bromide
PBS	phosphate-buffered saline; ROS, reactive oxygen species
SDS	sodium dodecyl sulfate
TBST	Tris-buffered saline containing 0.05% Tween 20
