# Supplementary material for: A Flavone Constituent from Myoporum bontioides Induces M-Phase Cell Cycle Arrest of MCF-7 Breast Cancer Cells
Source: Molecules. 2017 Mar 15;22(3):472. doi: 10.3390/molecules22030472 (PMC6155216; doi:10.3390/molecules22030472)
Supplement: Supplementary file 1 [file molecules-22-00472-s001.pdf]

Supplementary Materials: A Flavone Constituent from *Myoporum bontioides* Induces M-phase Cell Cycle Arrest of MCF-7 Breast Cancer Cells

Jing-Ru Weng, Li-Yuan Bai, Wei-Yu Lin, Chang-Fang Chiu, Yu-Chang Chen, Shi-Wei Chao, and Chia-Hsien Feng

Figure		Page
S1	^{1}H NMR spectrum (600 MHz, CD_3OD) of compound **1**	2
S2	^{13}C NMR spectrum (150 MHz, CD_3OD) of compound **1**	3
S3	HMQC spectrum of compound **1**	4
S4	HMBC spectrum of compound **1**	5
S5	NOESY spectrum of compound **1**	6

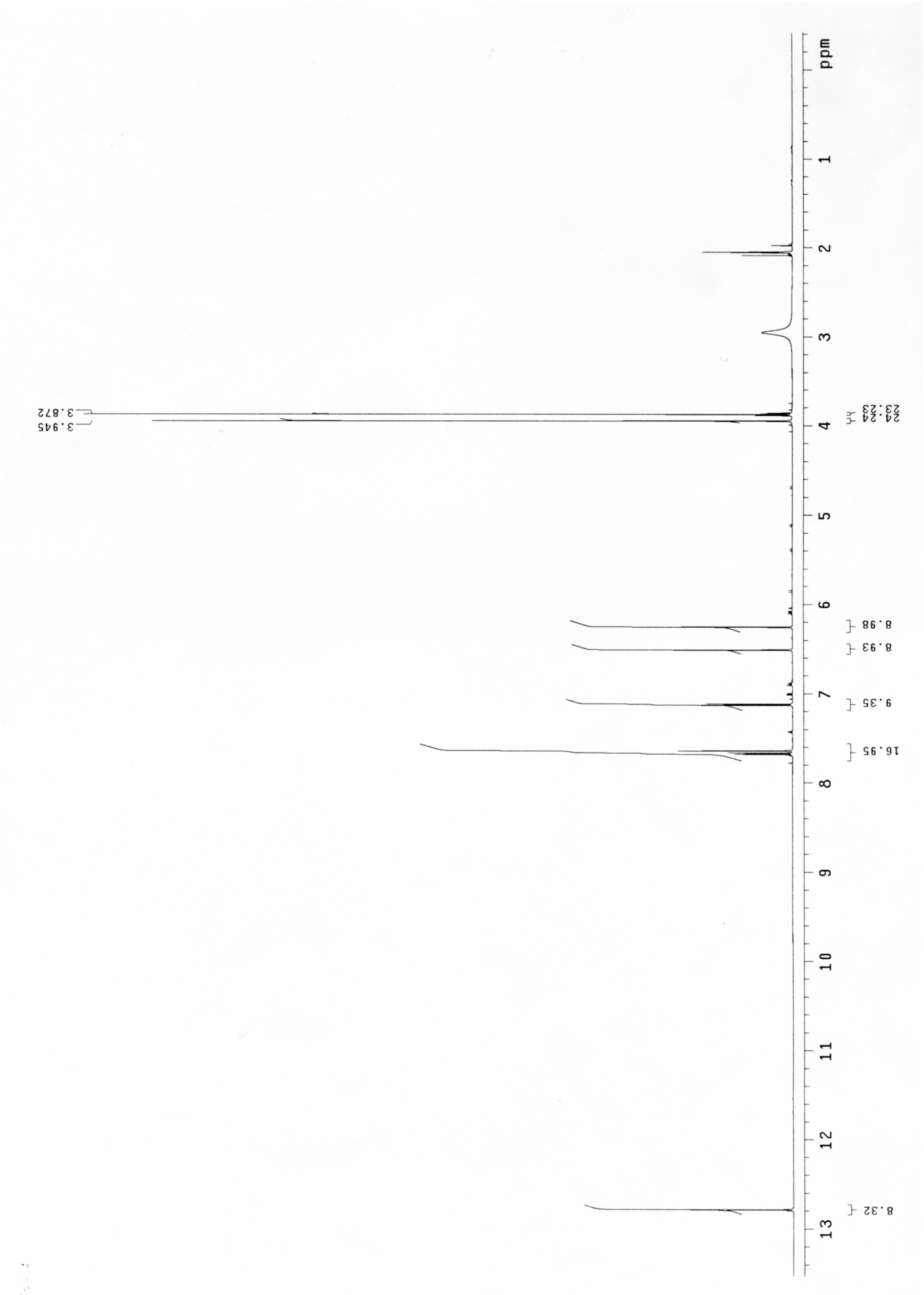

Figure S1. ^{1}H NMR spectrum (600 MHz, CD_3OD) of compound **1**

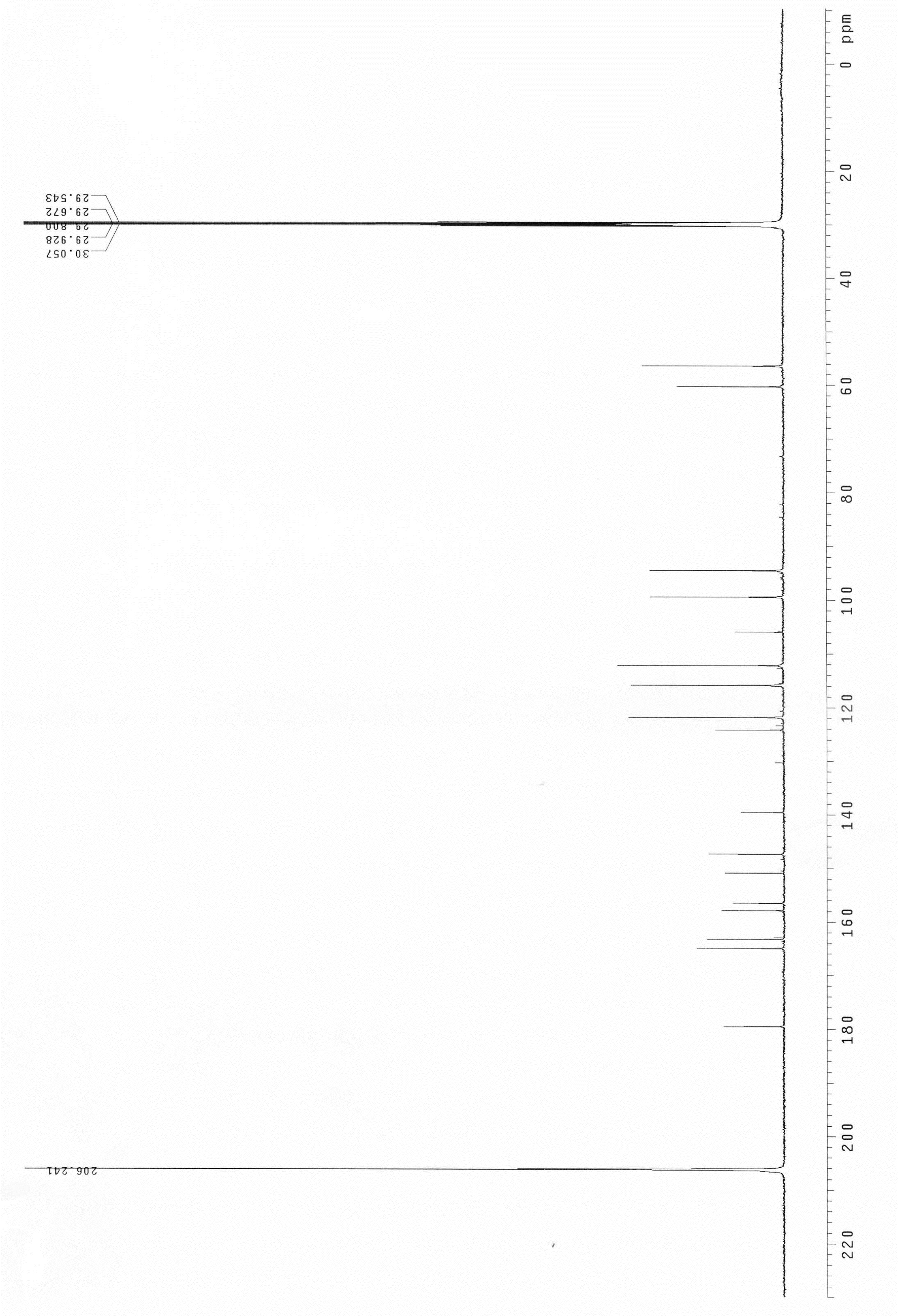

Figure S2. ^{13}C NMR spectrum (150 MHz, CD_3OD) of compound **1**

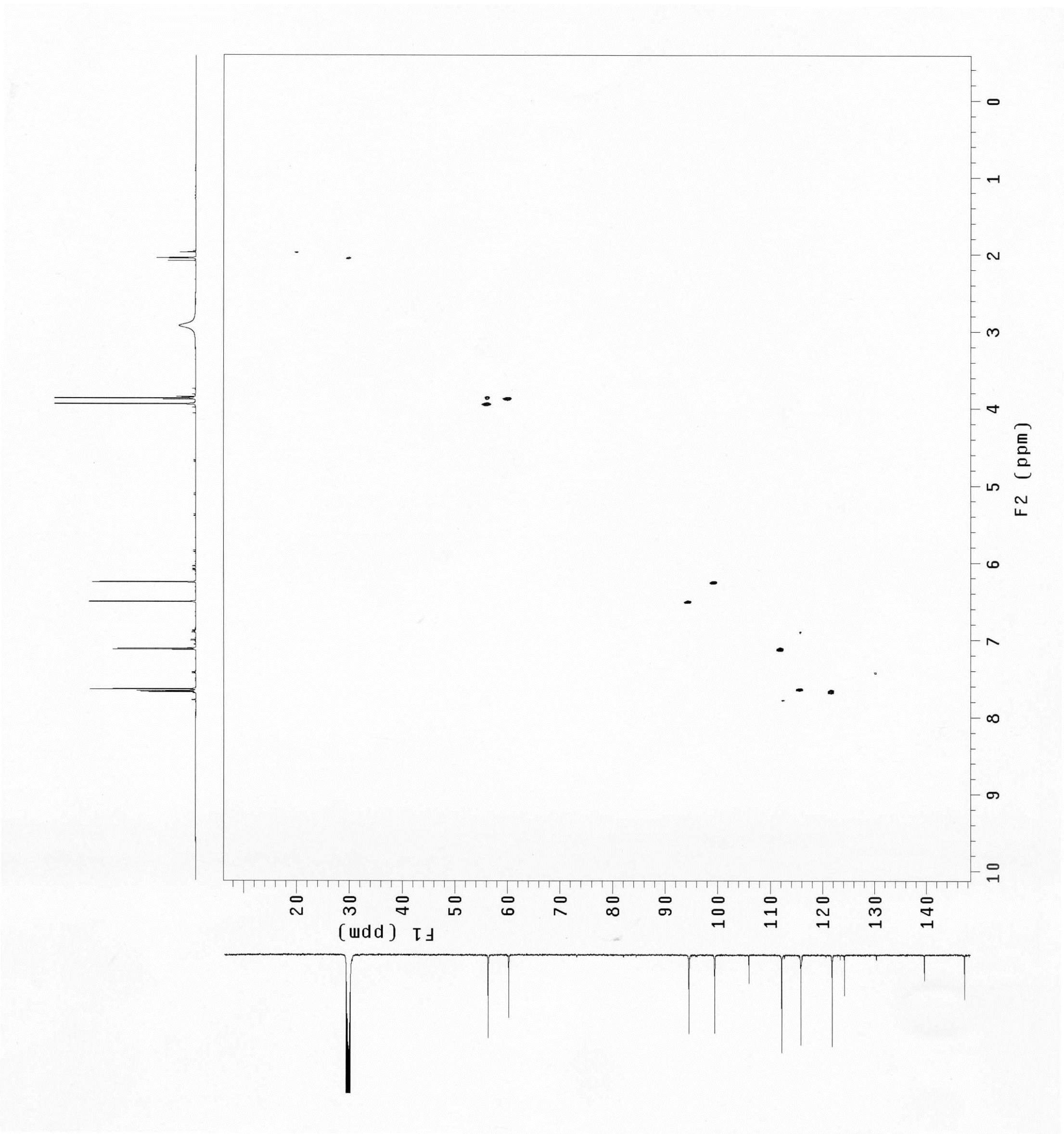

Figure S3. HMQC spectrum of compound **1**

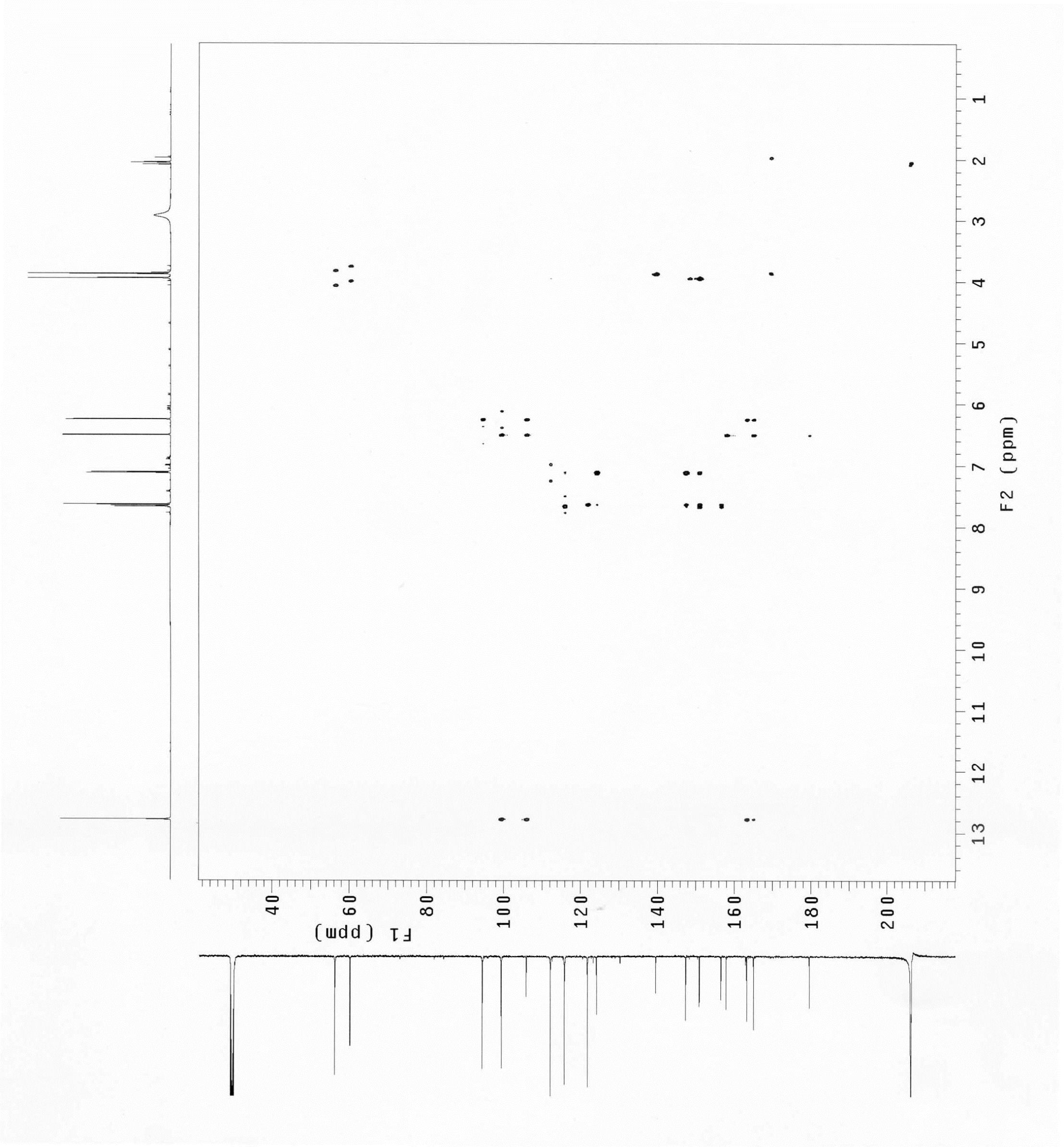

Figure S4. HMBC spectrum of compound **1**

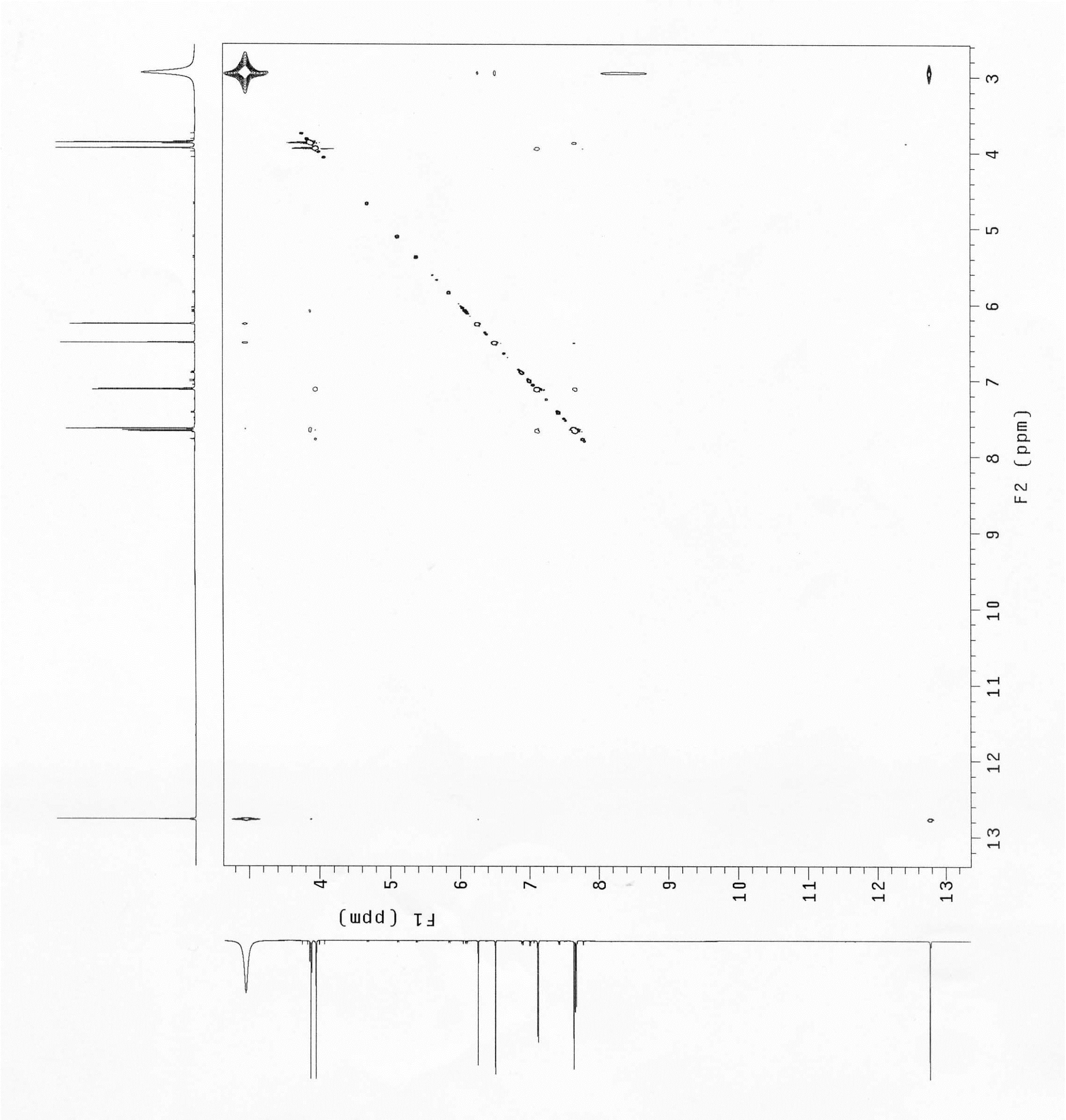

Figure S5. NOESY spectrum of compound **1**
